# Designing Novel Therapies to Mend Broken Hearts: ATF6 and Cardiac Proteostasis

**DOI:** 10.3390/cells9030602

**Published:** 2020-03-03

**Authors:** Erik A. Blackwood, Alina S. Bilal, Winston T. Stauffer, Adrian Arrieta, Christopher C. Glembotski

**Affiliations:** Department of Biology, San Diego State University Heart Institute, San Diego State University, San Diego, CA 92182, USA; eblackwo@alumni.nd.edu (E.A.B.); asusanab@gmail.com (A.S.B.); wstauffe@gmail.com (W.T.S.); aarrieta1335@gmail.com (A.A.)

**Keywords:** ATF6, cardiac myocyte, hypertrophy, proteostasis, small molecule, therapy, unfolded protein response (UPR), transcriptional regulation, cardiomyopathy

## Abstract

The heart exhibits incredible plasticity in response to both environmental and genetic alterations that affect workload. Over the course of development, or in response to physiological or pathological stimuli, the heart responds to fluctuations in workload by hypertrophic growth primarily by individual cardiac myocytes growing in size. Cardiac hypertrophy is associated with an increase in protein synthesis, which must coordinate with protein folding and degradation to allow for homeostatic growth without affecting the functional integrity of cardiac myocytes (i.e., proteostasis). This increase in the protein folding demand in the growing cardiac myocyte activates the transcription factor, ATF6 (activating transcription factor 6α, an inducer of genes that restore proteostasis. Previously, ATF6 has been shown to induce ER-targeted proteins functioning primarily to enhance ER protein folding and degradation. More recent studies, however, have illuminated adaptive roles for ATF6 functioning outside of the ER by inducing non-canonical targets in a stimulus-specific manner. This unique ability of ATF6 to act as an initial adaptive responder has bolstered an enthusiasm for identifying small molecule activators of ATF6 and similar proteostasis-based therapeutics.

## 1. Introduction

Cardiovascular disease (CVD) accounts for one in every three deaths in the US [1]. While various etiologies may contribute to the progression of CVD, they are generally associated with pathological left ventricular hypertrophy. Believed to be initially an adaptive compensatory response to maintain cardiac function and decrease ventricular wall tension, pathological cardiac hypertrophy can lead to a maladaptive remodeling of the heart during which there is thinning of the myocardium, chamber dilatation and a reduction in cardiac output and contractility, leading to eventual heart failure [2,3]. Despite improvements in clinical management, heart failure rates continue to represent the fastest-growing subcategory of CVD in an increasingly aging population [4,5,6,7], accounting for more than 500,000 deaths per year and resulting in an incredible economic impact of 100 billion USD per year [7,8,9]. While palliative measures are available and prescribed for patients to treat the symptoms associated with heart failure, aside from a heart transplant there is no clinically available curative therapy [3]. Furthermore, progress in therapeutic intervention is hindered by the lack of understanding of the molecular mechanisms underlying the pathophysiology of CVD and heart failure.

Cardiac hypertrophy requires an increase in protein synthesis in cardiac myocytes, much of which is responsible for the sarcomere growth necessary to maintain or improve global cardiac contractile function [10,11]. This net increase in protein is determined primarily by the rates of the synthesis, folding and degradation machinery to allow for homeostatic growth without affecting the functional integrity of cardiac myocytes, as misfolded proteins can be toxic [12,13,14,15]. Thus, the protein-folding load must equal that of the protein folding capacity to avoid toxic accumulation of misfolded proteins (proteostasis) [16,17,18]. Studies in both animal models and patients support imbalanced proteostasis as a primary driver of CVD and heart failure [11].

Proteostasis is maintained by intracellular pathways that coordinate protein synthesis and folding with the degradation of misfolded, potentially toxic proteins [19,20]. A majority of this protein synthesis occurs at the endoplasmic reticulum (ER), making it a major site of protein quality control [21]. Imbalances in proteostasis cause or exacerbate numerous pathologies, spawning interest in the exogenous manipulation of proteostasis as a therapeutic approach for such diseases [22]. ER proteostasis is regulated by the unfolded protein response (UPR), a stress-responsive signaling pathway comprising three sensors/effectors of protein misfolding, PERK (protein kinase R [PKR]-like ER kinase), IRE1 (inositol requiring enzyme 1), and ATF6 (activating transcription factor 6α) [23]. Considerable evidence supports ATF6, a transcriptional regulator of ER proteostasis, as a viable therapeutic target for exogenous manipulation of proteostasis [24,25,26,27,28,29]. This review focuses on the therapeutic potential of ATF6 in maintaining cardiac myocyte proteostasis by inducing canonical and non-canonical gene targets in CVD and, more specifically, cardiac hypertrophy.

## 2. Cardiac Hypertrophy in Health and Disease

Cardiac myocytes, which comprise 85% of the heart mass, are responsible for generating the contractile force necessary for maintaining systemic blood flow of oxygen and nutrients [30,31]. The force-generating units of cardiac myocytes are tightly aligned sarcomeres that, in response to an increase in workload, grow via addition of sequential nascent sarcomeres in length and/or width, depending upon the nature of the stimulus [31]. Cardiac myocytes are uniquely susceptible to damage associated with chronic increases in workload or stress, due to their limited potential to proliferate [31]. For this reason, hypertrophic growth via increased protein synthesis in cardiac myocytes is the primary mechanism whereby the heart reduces ventricular wall stress [10,32]. This hypertrophic growth was seen by physicians as initially a compensatory response mechanism, as it develops in accordance with Laplace’s law, dictating that increases in pressure or volume load-induced tension in the heart must be offset by an increase in myocardial and ventricular wall thickness [33,34,35,36]. While ongoing studies are beginning to question this concept and the necessity for cardiac hypertrophy primarily in response to pathological stimuli [37], what is apparent is that the requisite increase in protein synthesis in any form of cardiac myocyte growth strains the protein-folding machinery in the heart. This strain must be abated for sustained cardiac function [12,13,14,15]. 

### 2.1. Developmental and Physiological Cardiac Hypertrophy

**Figure 1:**
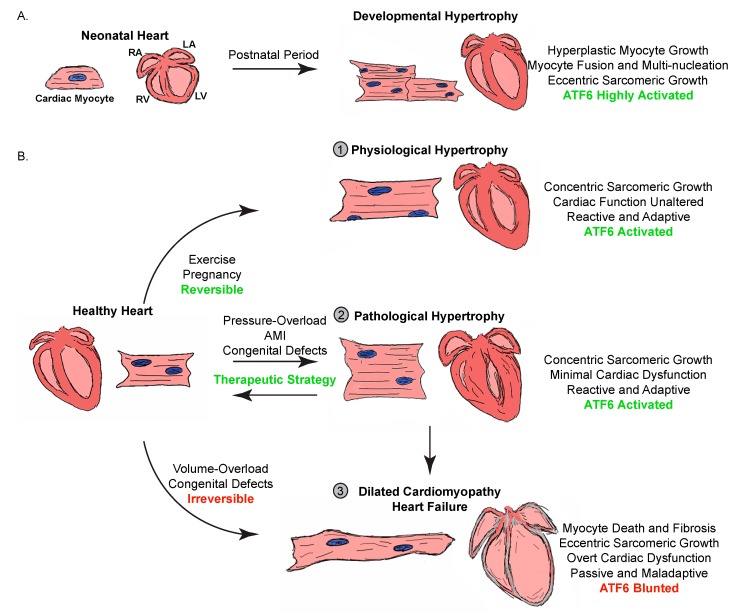
Types of cardiac hypertrophy. (**A**) Cross-section drawings are shown to demonstrate the growth of the heart during pre- and postnatal development. This developmental hypertrophy is depicted showing increases in both atrial (RA and LA) and ventricular (RV and LV) chamber blood volume (pink) and wall thickness (red areas are myocardium). Developmental cardiac hypertrophy from the neonatal stage to adulthood is characterized by both hyperplasia and hypertrophy, and since ATF6 is expressed in robust quantities during this period of development, the ER protein folding machinery is sufficient to support the protein-folding load. (**B**) The adult healthy heart undergoes three main types of cardiac hypertrophy: ① Physiological hypertrophy is an adaptive and reactive process of concentric growth in response to chronic exercise and pregnancy. ATF6 is robustly activated by this process, and the ER protein folding machinery is sufficient to support this form of hypertrophy. ② Pathological hypertrophy is considered an adaptive and reactive process of concentric growth in response to pressure-overload or AMI. In the acute compensatory stages of this concentric growth, ATF6 is robustly activated by this process, and the ER protein folding machinery is sufficient to support this form of hypertrophy. This form of cardiac hypertrophy is reversible and a potential target of ATF6-based therapeutics. ③ Dilated cardiomyopathy and heart failure are either a result of chronic pathological hypertrophy or congenital defects. This is a passive process characterized by chamber dilatation and cardiac myocyte apoptosis and fibrosis. ATF6 and the protein folding machinery are not sufficient at this stage of maladaptive growth.

Despite the connotation, a number of physiological conditions can provoke cardiac hypertrophy and dramatic changes to cardiac myocyte number and size, beginning with development [31]. As depicted in Figure 1A, the pre- and post-natal heart grows in cardiac myocyte number, or hyperplasia [38,39]. However, this replicative capacity is lost in as little as four days after birth [40], and continued increases in heart mass to meet an increasing circulatory demand are achieved through hypertrophic growth of preexisting cardiac myocytes [41,42]. This state of cell-cycle arrest of post-natal cardiac myocytes is associated with maturation of the gene programs governing isoforms of contractile proteins and calcium handling proteins, as well as a shift in the preferred energy source for cardiac myocytes, from anaerobic glycolysis to an oxygen-dependent mitochondrial oxidative phosphorylation [43]. While the mechanism is still unclear, this adaptation is greatly influenced by nutritional, hemodynamic, humoral, and even oxygen tension changes from the environment in utero [43]. Upon the cessation of hyperplastic growth, the heart hypertrophies in an eccentric manner characterized by an overall increase in cardiac mass, as well as chamber volume. Due to this abrupt dependence on hypertrophic growth of cardiac myocytes, it is imperative for the protein-folding machinery to meet the demands of increases in protein synthesis. In fact, neonatal and adolescent cardiac myocytes exhibit a robust adaptive UPR and ATF6 activity, as evidenced by the finding that the expression of many of the components of the canonical gene panel regulated by ATF6 is relatively high in the young heart, compared to the adult and aged heart [44,45].

Normal growth of the heart during adolescence and adulthood is driven by physiological cardiac hypertrophy, a reactive growth occurring as a direct response to extrinsic stimuli necessitating an increase in cardiac output (Figure 1B, Form 1) [37,43,46]. Similar to postnatal hypertrophy, pregnancy, and prolonged exercise induce greater circulatory demands that inevitably drive a concentric growth of the heart, characterized by a relative increase in wall thickness and cardiac mass, but little or no change in chamber volume [43,46]. Coordinately, while physiological stimuli elicit a concentric manner of cardiac myocyte hypertrophy, the sarcomeric growth in diameter and length is nearly proportional. Ironically, in the 19th century, this form of cardiac hypertrophy was thought to be pathological in nature and a result of overexertion, even being coined as “athlete’s heart” [47]. Indeed, while well trained athletes can exhibit increases in cardiac mass by up to 60%, the transient nature of this form of heart growth, which can rapidly reverse upon hemodynamic unloading, was not conceived of until later on [48,49]. It is now known that growth of the myocardium as a result of pregnancy or exercise is not associated with heart failure progression and is adaptive in nature, due to the sustained or even increased cardiac output [43]. 

While adaptive, the increase in protein folding associated with physiological hypertrophy would be expected to strain the proteostasis network. However, studies have shown that not only is ATF6 activated to regulate an adaptive gene panel allowing for continued growth [50], but also, exercise can revert many of the age-related symptoms leading to CVD, such as attenuated accumulation of misfolded protein aggregates within cardiac myocytes [51].

### 2.2. Pathological Cardiac Hypertrophy

Pathological cardiac hypertrophy is a reactive response to either genetic or environmental/habitual diseases that primarily affect cardiac myocytes [31,37]. The two major effectors of pathological cardiac hypertrophy are biomechanical stress and neurohumoral factors, both increasing cardiac workload. Subsequently, intracellular signaling cascades associated with an increase in protein synthesis are activated, and thus increase protein folding demand [37]. If proteostasis is not maintained throughout this growth, the integrity of the cardiac myocyte structure and contractile function is severely impaired, leading to eventual heart failure (Figure 1B, Forms 2 and 3). Hypertension and pressure-overload is the most important risk factor for heart failure, and data from the Framingham Heart Study demonstrated that the severity of hypertension and coordinate pathological cardiac hypertrophy is a prognostic indicator of heart failure risk [37,52,53]. Traditionally, hypertensive and pressure- or volume-induced cardiac hypertrophy is viewed as an adaptive response characterized by concentric growth of the heart and cardiac myocytes, as evidenced by a relative increase in wall thickness and mass without affecting chamber volume, as well as sarcomeric growth in diameter as opposed to length [31,37]. At this stage, there is minimal effect on cardiac output, and many of the symptoms are reversible, making it a prime target of therapeutic intervention. However, with prolonged stress, the heart undergoes an irreversible state of decompensation, characterized by chamber dilatation due to cardiac myocyte death, fibrotic remodeling, and immune cell infiltration, as well as a decreased cardiac output and compliance, leading to inevitable heart failure [37]. The dangers and pathogenesis of even early-stage concentric cardiac hypertrophy in response to hypertension have been noted as early as the 19th century by William Osler and physicians observing the broken nature of this compensatory phase of remodeling that is coordinate with degenerating myocardium [54]. Late-stage decompensation impairs excitation-contraction coupling, thus increasing the risk of malignant arrythmia and death [37]. Continued research efforts are aimed at dissecting the adaptive signaling mechanisms underlying the initial compensatory phases of cardiac hypertrophy that decrease ventricular wall stress in accordance with Laplace’s law, while negating the maladaptive features associated with decompensation. 

Pathological hypertrophy can also be secondary to chronic conditions not directly linked to hemodynamic stress, such as coronary artery disease (CAD) and ischemic heart disease or injury, including acute myocardial infarction (AMI), where thrombotic coronary artery occlusion causes rapid, irreparable ischemic injury to the heart [55,56,57,58,59]. Much of the damage associated with AMI occurs from reperfusion injury, which, ironically, results from the only treatment option, primary percutaneous coronary intervention, or coronary angioplasty [60]. While reperfusion limits ischemic injury, which would otherwise be fatal, coronary angioplasty causes a rapid generation of reactive oxygen species (ROS) leading to cardiac myocyte death, due mainly to impaired proteostasis [61,62]. Since cardiac myocytes in adults are incapable of regeneration, AMI damage is essentially permanent and can set in motion a pathological remodeling of the heart, culminating eventually in heart failure and arrhythmogenesis [3].

More recently, a less well-defined form of pathological cardiac hypertrophy, heart failure with preserved ejection fraction (HFpEF), has emerged as an important pathology due to its clinical prevalence and association with ever increasing metabolic diseases [63]. HFpEF is characterized by concentric cardiac hypertrophy without overt systolic impairment, and is associated with a patient population diagnosed with the comorbidities of obesity, type II diabetes mellitus and chronic hypertension [63,64,65]. While initially thought of as a form of diastolic heart failure, HFpEF is further characterized by impaired active myocardial relaxation and increased passive stiffness, as well as increased pulmonary capillary wedge pressures that can rise to levels above that of even hypertensive patients, or those with aortic stenosis [66,67]. However, underappreciated until more recently is the contribution of non-cardiac myocytes in the heart to the progression of HFpEF, namely endothelial cells, which lead to derangement of nitric oxide bioavailability, thus leading to cardiac myocyte hypertrophy subsequent to impaired Ca^2+^ handling [63]. HFpEF has also been directly linked to impaired proteostasis, as extracellular cardiac amyloid deposition and nitrosative stress strain the proteostasis network, resulting in protein damage that activates the adaptive UPR response pathway [68,69].

Given that the increase in the demands on the protein-folding machinery that is associated with cardiac hypertrophy, which has been shown to activate ATF6 and the adaptive UPR in order to maintain proteostasis and heart function, ATF6 becomes a potential therapeutic target for mitigation of the proteotoxicity associated with numerous models of CVD.

## 3. The ER Unfolded Protein Response in Cardiac Myocyte Proteostasis

### 3.1. Proteostasis

The increase in protein folding demand associated with nascent protein synthesis occurring during cardiac hypertrophy puts a strain on the global cellular framework responsible for balancing proteostasis, which is necessary to allow for proper cardiac myocyte growth and is critical to maintaining cardiac function [10,11,12,13,14,15]. Proteostasis is maintained by cellular networks that balance protein synthesis with proper folding, trafficking, and degradation [15,70]. Imbalances in this cellular framework can lead to the accumulation of proteotoxic misfolded protein aggregates and proteinopathies, contributing to a multitude of systemic diseases including CVD and cardiomyopathies, eventually leading to heart failure [71,72,73,74,75,76]. In addition to CVD, impaired proteostasis has been intimately linked to aging-related diseases thought to be a result of genetic and environmental derailment of the integrity of the proteome, fundamental to the progression of many neuronal-based diseases such as Alzheimer’s, Parkinson’s, and Huntington’s disease [17].

While the proteostasis framework encompasses numerous proteins comprising chaperones, foldases, and scaffolds (assisting in the proper folding and refolding of proteins), the focus of many research efforts aimed at designing proteostasis-based therapeutics has been on the ubiquitin-proteasome system (UPS) responsible for the clearance of aggregation-prone misfolded proteins [30]. In fact, considering that as many as 30% of nascent proteins during cardiac hypertrophy never reach their final folded confirmations, and therefore, must be degraded either concurrently or very soon after translation, emphasizes the critical nature of the UPS in maintaining proteostasis [77,78]. Furthermore, the majority of nascent proteins made during cardiac hypertrophy include sarcomeric proteins, calcium-handling proteins, or receptors destined for the sarcolemma, implicating the importance of not only the temporal kinetics of protein degradation, but also the spatial location of proteosomes relative to translational “hot-spots” for maintenance of proteostasis during cardiac myocyte growth.

### 3.2. ER Associated Degradation

As many as 40% of nascent proteins are translated on ER-associated ribosomes, including secreted and membrane proteins requiring transit across the ER membrane during translation in conjunction with either co- or post-translational folding prior to terminal trafficking [79]. Due to the high volume of protein translation associated with the ER, the ER maintains proteostasis in part by eliminating terminally misfolded or excess proteins via the evolutionarily conserved ER-associated degradation (ERAD) pathway [80]. ER luminal chaperones recognize these potentially proteotoxic hazards, and ERAD is initiated via retrotranslocation of polypeptides from the ER membrane or lumen to the cytosol, a process requiring the AAA+-type ATPase valosin-containing protein (VCP) to be recruited to the ER surface via a VCP-adaptor protein (e.g., Vimp) [81,82]. This ‘ratchet’ effect for extracting proteins from the ER then allows ER-transmembrane E3 ubiquitin ligases (e.g., Hrd1) to mark them for proteasome-mediated degradation [83,84]. ERAD complex constituents recognize a vast array of misfolded proteins and while the mechanism of substrate recognition is still unclear, it is known that the constituents of the complex dictate a degree of substrate specificity. Thus, specific VCP-adaptor proteins, such as Vimp, Derlin1, or Ufd1, that separately coordinate ERAD complex formation could recognize specific substrates.

### 3.3. ER UPR

Cardiac myocytes comprise the majority of the cellular mass of the myocardium and, within the ventricles, function primarily as the contractile unit required for pumping oxygenated blood into the circulation. Because of this dominant role, many studies have focused on sarcomeric and calcium-handling proteins as part of the contractile calcium handling process vital for cardiac myocyte contractility. However, the majority of sarcolemmal and secreted proteins integral to maintaining proper cardiac function via proper excitation-contraction coupling and endocrine/paracrine signaling under physiological conditions, as well as during cardiac hypertrophy, are made in the ER [85,86]. Furthermore, many post-translational modifications vital for proper protein function occur in the ER, namely glycosylation, disulfide bond formation, and proteolytic processing [87,88]. Thus, in the heart, etiologies related to CVD, including pressure- or volume-overload, and AMI place high demands on the ER protein folding machinery to maintain ER proteostasis.

Under conditions in which the protein folding demand outweighs the capacity of the ER protein folding machinery, such as during hypertrophic cardiac myocyte growth, the UPR is activated [14]. Acute activation of the UPR balances proteostasis and maintains viability and function of cardiac myocytes primarily via genetic modification. The PERK, IRE1, and ATF6 arms of the UPR overlap to an extent, however, they each confer individualistic downstream signaling cascades aimed at restoring proteostasis [23].

The PERK branch of the UPR functions primarily to phosphorylate the translation initiation factor, eIF2α on Ser-51 resulting in global arrest of 5′ cap-dependent protein translation in an attempt to acutely decrease the protein folding load [89]. Phosphorylation of eIF2α allows for the continued translation of a subset of mRNAs required for adaptive ER proteostasis. The role of PERK in CVD has been studied using a mouse model in which PERK has been selectively deleted in cardiac myocytes using a conditional gene targeting approach. These studies revealed that in a model of pressure overload-induced heart failure, PERK was critical for attenuating fibrotic remodeling associated with excessive cardiac myocyte apoptosis and immune cell infiltration [90].

IRE1 functions primarily as an endonuclease responsible for splicing the Xbp1 mRNA to a splice variant (Xbp1_s_) encoding an active transcription factor [91]. The nuclease ability of IRE1 can contribute to decreasing the protein folding load of the ER via cleavage of mRNAs localized to the ER membrane that are not critical for the adaptive UPR. This process is called regulated Ire1-dependent decay (RIDD) [92]. For the most part, the gene program induced by XBP1_s_ overlaps to an extent with ATF6, comprised mostly of chaperones and ERAD components. However, studies using a transgenic mouse model to overexpress XBP1_s_ specifically in cardiac myocytes have demonstrated that XBP1_s_ confers protection against AMI injury and post-AMI cardiac remodeling by transcriptionally regulating non-canonical targets that are key components of the hexosamine biosynthetic pathway [93]. More recently, it was shown that XBP1_s_ is protective in a novel murine model of HFpEF via its ability to induce those same proteins involved in O-GlcNAcylation [69]. XBP1_s_ has an additional role in regulating cardiac hypertrophy in response to pressure-overload via transcriptional induction of FKBP11, and thus regulating mTORC1 activity [94].

## 4. ATF6 and Proteostasis in the Heart

Upon sensing an increase in protein folding demand, ATF6 acts as the primary adaptive responder that is liberated from the ER to act as an active transcription factor regulating a gene program that fosters the maintenance of proteostasis in cardiac myocytes [95,96,97]. In response to CVD and various hypertrophic growth stimuli in the heart, ATF6 is activated, which allows for homeostatic growth and regulation of proteostasis via transcriptionally inducing gene targets that function to either temper protein synthesis, fold nascent proteins, or degrade misfolded and surplus proteins [50].

### 4.1. ATF6 Activation

**Figure 2:**
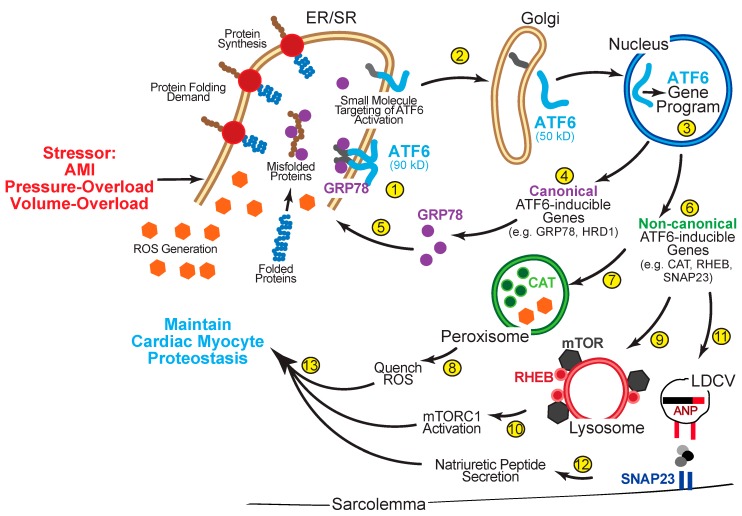
ATF6 activation and gene program induction. ① In its inactive state, ATF6 is a 90kD ER transmembrane protein that is anchored in the membrane in oligomers via GRP78 and intermolecular disulfide bonding. ② Upon stressors, like cardiac hypertrophy that increases protein synthesis and the protein folding demand or AMI that elevates cellular levels of reactive oxygen species (ROS), GRP78 dissociates from the ER luminal domain of ATF6 and the disulfide bonds are reduced allowing monomerization of ATF6, which allows the 90 kD form of ATF6 to translocate to the Golgi, where is it cleaved by S1P and S2P to liberate the N-terminal approximately 400 amino acids (50 kD) of ATF6 from the ER membrane. It is this unique sequence of activation steps that open a window of small molecule targeting and discovery of ATF6-based therapeutics. ③ The clipped form of ATF6 has a nuclear localization sequence, which facilitates its movement to the nucleus where it binds to specific regulatory elements in ATF6-responsive genes, such as ER stress response elements (ERSEs), and induces the ATF6 gene program. ④ The canonical ATF6 gene program comprises genes that encode proteins that localize to the ER, such as the chaperone, GRP78, or components of ERAD, HRD1 ⑤, where they fortify ER protein folding. ⑥ The non-canonical ATF6 gene program comprises genes that encode proteins not typically categorized as ER stress-response proteins, many of which localize to regions of the cell outside the ER. ⑦ Catalase is a potent antioxidant that localizes to the lumen of peroxisomes where it functions to ⑧ quench ROS. ⑨ Rheb is a small GTPase located on the surface of lysosomes that when bound to mTOR, ⑩ promotes mTORC1 activation and sustains protein synthesis. ⑪ SNAP23 is a t-SNARE protein crucial for ⑫ secretion of natriuretic peptides via large dense-core vesicles (LDCV) in response to hemodynamic stress. ⑬ Both the canonical and non-canonical ATF6 gene programs coordinate to maintain cardiac myocyte proteostasis.

The activation process of ATF6 is a tightly regulated process and, in the absence of proteostatic imbalance, exists as a 90 kD ER transmembrane protein (Figure 2, Step 1) [98]. As a primary sensor/effector of the UPR, ATF6 recognizes an increase in protein folding demand brought about by stressors such as pressure- or volume-overload, or an accumulation of misfolded proteins as occurs during an AMI. While the mechanism by which stimulus-specific stressors are differentially recognized and integrated by ATF6 remains unclear, the primary activation process is mediated by the ER chaperone, GRP78, as well as protein disulfide isomerases (PDIs) [97,99]. ATF6 is kept inactive and retained in the ER via the binding of GRP78 to the ER-luminal domain of ATF6 cloaking a Golgi localization sequence [100,101]. Secondarily, inactive ATF6 exists in an oligomeric state via intermolecular disulfide bonding regulated by PDIs, such as PDIA5, and only upon the reduction of the disulfide bonds [97], and subsequent dissociation of GRP78 [100,101], can ATF6 monomerize and translocate to the Golgi, where it is cleaved via regulated intramembrane proteolysis by S1 and S2 proteases (Figure 2, Step 2) [102]. This proteolysis liberates an N-terminal 50 kD fragment of ATF6 that is able to freely translocate to the nucleus via a nuclear localization sequence (Figure 2, Step 3) [98] where it recognizes and binds to promoter regions containing canonical ATF6 binding motifs, such as the ER stress element (ERSE) [95]. For the most part, ATF6 is known for regulating canonical gene targets as part of an adaptive panel destined for the ER and designed to regulate ER protein folding (Figure 2, Steps 4 and 5) comprised of ER-resident chaperones (e.g., GRP78), PDIs, and ERAD components (e.g., HRD1) [11,14,16,18]. However, recent studies have illuminated a remarkable ability of ATF6 to induce non-canonical gene targets that were not previously known to be genes related to the UPR nor reside in the ER, but instead are induced by ATF6 in a stimulus-specific manner and localize to specific regions of cardiac myocytes including the lysosome [50], peroxisome [28] and sarcolemma (Figure 2, Step 6) [103]. 

### 4.2. Early Findings of ATF6 in the Heart

The initial studies of ATF6 in the heart came about somewhat serendipitously. During the investigation of gene induction in response to hypertrophic stimuli, it was discovered that ATF6 was a requisite binding partner of Serum response factor (SRF) allowing for subsequent recognition and binding to canonical SRF promotor motifs, serum response elements (SREs) and thus allowing for proper cardiac myocyte growth. This was a bit of foreshadowing, and the first indication that ATF6 could be required for cardiac hypertrophy [104].

Following these initial discoveries in cultured cardiac myocytes, the fascinating biochemistry of ATF6 quickly attracted attention, which led to considerable continued investigation. While at this time, it was still unclear if pathophysiological stimuli activated ATF6, what was apparent was the transient nature of its activity. Once activated, ATF6 exhibits a robust influence on gene induction followed by its own rapid degradation [105]. In fact, the half-life of ATF6 was noted to be so short, it was actually an initial impediment for continued experimentation until proteasome inhibitors were used to decrease its degradation sufficiently to allow for detection. The rapid half-life was found to be directly correlated to its transcriptional induction capacity as domain mapping of ATF6 led to the identification of a unique 8 amino acid sequence shared with the rapidly-degraded viral transcription factor, VP16, and deletion of this motif not only attenuated transcriptional activity, but prolonged the half-life of ATF6 [105,106,107]. Therefore, it appears that ATF6 was designed to be a rapid and transient adaptive response transcription factor, reasons for which are still unclear.

### 4.3. ATF6 Regulates an Adaptive Gene Panel in the Heart

In an attempt to delineate a functional role for ATF6 and proteostasis in the heart, the first ATF6 transgenic mice were generated. These mice were designed so that ATF6 could be selectively expressed and activated ATF6 [27]. As researchers were keenly aware of the importance of the transient nature of ATF6 activity, this transgenic mouse was designed such that ATF6 could be conditionally activated by fusing activated ATF6 to the mutant mouse estrogen receptor (MER), unmasking of the ATF6 transactivation domain upon the introduction of tamoxifen. Upon initial study and characterization, it was discovered that similar to endogenous ATF6 in model cell lines, the ATF6-MER was rapidly degraded upon activation and was the first in vivo evidence of the “degraded-when-active” property of ATF6 [27]. Subsequent microarray analysis of ventricular extracts identified approximately 400 genes to be regulated by ATF6 using this transgenic mouse, the majority of which make up a canonical adaptive gene profile of ER-targeted proteins to regulate ER-protein folding [108].

While at this time, it was becoming more apparent that a number of pathological conditions, including pressure-overload, ischemia, and AMI could cause an imbalance in proteostasis and activate the UPR, many studies emerged focusing on a role of downstream targets of ATF6 in CVD. Hrd1, a ubiquitin E3 ligase and integral for the ERAD system, was demonstrated to modulate cardiac hypertrophy and to restore cardiac function in a pressure-overload model of heart failure, presumably by enhancing the degradation of proteotoxic misfolded proteins and thus promoting cardiac myocyte viability [44]. A separate ATF6-inducible target that contributes to the translocation of misfolded proteins out of the ER in the ERAD system, Derlin3, was shown to be protective in cultured cardiac myocytes subjected to in vitro ischemia-reperfusion (I/R) and to enhance ERAD of terminally misfolded proteins in an ATF6-dependent manner [109]. Additional canonical targets of ATF6 identified in the ATF6-MER hearts included the protein disulfide isomerase, PDIA6 [110], which was shown to confer protection against in vitro I/R, as well as ER-resident chaperones, MANF and GRP78 [108,111,112]. MANF and GRP78 became interesting targets of ATF6, as they not only were known to enhance ER protein folding, but despite both having ER-retention motifs anchoring them inside the ER, were demonstrated to be actively secreted from cultured cardiac myocytes upon only select stimuli known to deplete ER Ca^2+^ [112]. Subsequent to their trafficking and secretion, MANF and GRP78 function adaptively in the extracellular matrix or at the sarcolemma via maintaining cardiac myocyte proteostasis [113]. Furthermore, GRP78 has drawn attention as a potential therapeutic target as an adaptive response protein conferring protection in the setting of AMI via activating the pro-survival kinase, Akt [114], and enhance the cardiac hypertrophic response by activating the pro-growth transcription factor, GATA-binding protein 4 (GATA4) [115].

## 5. ATF6 Is an Adaptive Responder in CVD via Regulating Non-Canonical Genes

### 5.1. ATF6 Is Protective in Models of Acute Myocardial Infarction

Studies highlighting the protective roles of canonical gene targets of ATF6 have further fortified interest in pursuing ATF6-based therapeutics in clinically relevant disease models, namely AMI and pathological cardiac hypertrophy. Using the ATF6-MER model described above, it was discovered that ATF6 could confer protection against a model of I/R injury using an ex vivo Langendorff perfused heart system [27]. ATF6 activation blunted infarction and preserved cardiac contractile function in an acute injury model being the first report demonstrating activated ATF6 could exert widespread protective effects in any tissue, in vivo. Coordinately, additional studies demonstrated that using the ATF6-MER model in which the transgene was conditionally expressed in mouse forebrain neurons mitigated infarct size during an acute murine ischemic stroke model via occlusion of the middle cerebral artery (MCAO) [29].

Despite several studies demonstrating the efficacy of ATF6 in mitigating AMI injury, the mechanism of how ATF6, an ER-resident transcription factor, could protect from reperfusion damage associated with AMI or stroke, most of which is caused by oxidative stress and ROS generated by mitochondria, remained elusive. Accordingly, to address this mechanism, recent studies used either a mouse model where ATF6 had been globally deleted (ATF6 KO) [28] or generated a mouse model in which ATF6 is conditionally deleted only in cardiac myocytes (ATF6 cKO) [116]. Transcript profiling of ATF6 transgenic and ATF6 cKO mice revealed that in addition to genes encoding proteins that constitute the ER protein-folding machinery, ATF6 induces genes that encode proteins that do not even reside in the ER. One such group of genes encodes antioxidant proteins that reside outside the ER, including peroxisomal catalase (Figure 2, Step 7). This was a surprise because it was the first time antioxidant genes were shown to be induced by ATF6 in any cell or tissue type, and the first study to identify ATF6 as a direct transcriptional inducer of the catalase gene. This study demonstrated that it is through this non-canonical role that the scope of ATF6 action extends well beyond canonical UPR gene program to include proteostasis regulatory pathways, such as antioxidants, that can protect the heart from AMI damage (Figure 2, Step 8).

### 5.2. ATF6 Is Required for Cardiac Myocyte Hypertrophy 

**Figure 3:**
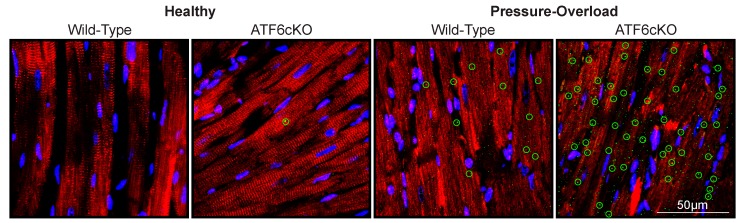
Effect of cardiac myocyte-specific ATF6 deletion in mouse hearts subjected to acute pressure-overload. Mice in which ATF6 has been selectively deleted in cardiac myocytes were subjected to an acute model of pressure overload-induced cardiac hypertrophy. Confocal immunocytofluorescence microscopy analysis of mouse heart sections is shown for a cardiac myocyte marker, Cardiac troponin T (red), protein amyloid oligomers (green), and nuclei (blue). The accumulation of misfolded protein aggregates indicates the necessity of ATF6 to support the protein folding load during concentric cardiac hypertrophy.

In considering other possible non-canonical roles for ATF6, a recent study set out to test whether the increase in protein synthesis and protein folding demand that occur during cardiac hypertrophy poses a challenge to the protein folding machinery, resulting in ATF6 activation [50]. Indeed, ATF6 was activated in mouse hearts subjected to conditions that mimic not only pathological (pressure-overload), but also physiological (exercise) cardiac myocyte growth. Using ATF6 cKO mice, it was demonstrated that ATF6 is required for heart growth and for maintaining cardiac function under both conditions. Furthermore, ATF6 was necessary to prevent the accumulation of misfolded proteotoxic aggregates during pressure overload-induced pathological cardiac hypertrophy (Figure 3). The finding that ATF6 was required for exercise-induced physiological cardiac hypertrophy was surprising, as it’s a reactive growth process known to, if anything, decrease the accumulation of misfolded proteins, thus linking ATF6 activation primarily to the increase in protein synthesis. While many ATF6 regulated genes may contribute to this effect, RNAseq and ChIP analysis identified one gene that had not previously been shown to be regulated by ATF6, i.e., Rheb. Rheb is an activator of mTORC1, a major inducer of protein synthesis and subsequent myocyte growth during pathological and physiological hypertrophy [117]. While not previously studied in the heart, constitutive mTORC1 activation via inhibition of the tuberous sclerosis complex (TSC1/2), the negative regulator of Rheb, has been shown to activate the UPR in model cell lines [118]. Rheb expression increased and mTORC1 was activated during both physiological and pathological hypertrophy, but not in ATF6 cKO mouse hearts. AAV9-mediated ectopic expression of Rheb restored cardiac myocyte growth to ATF6 cKO hearts. Similar results were found in a more recent publication where blunted pressure overload-induced cardiac hypertrophy and an accelerated progression to heart failure in ATF6 cKO mice [119]. Thus, ATF6 plays a previously unappreciated role in cardiac hypertrophy via inducing the non-canonical target, Rheb (Figure 2, Steps 9 and 10).

### 5.3. ATF6 Enhances Natriuretic Peptide Secretion and Hemodynamic Balance

Recent unpublished work has found yet another non-canonical target of ATF6 in a model of volume overload-induced cardiac hypertrophy [103]. When mice were subjected to a high salt diet, which is known to increase cardiac afterload primarily via increased blood volume, ATF6 cKO mice displayed advanced cardiovascular pathology characterized by hemodynamic imbalance and decreased cardiac compliance. The heart responds to high salt-induced hypertension by increasing the secretion of atrial natriuretic peptide (ANP) from atrial myocytes. In this regard, ANP increases natriuresis and diuresis and thus, decreases blood pressure [120,121,122]. While it was found that ATF6 did not affect cellular levels of ANP in atrial myocytes, ATF6 was found to be required for the regulated secretion of ANP from cultured atrial myocytes, as well as from mouse hearts. Mechanistically, ATF6 was shown to induce genes encoding proteins required for secretory granule exocytosis, including the t-SNARE, Snap23 [123,124]. Ectopic expression of Snap23 in the setting of ATF6 loss-of-function restored regulated ANP secretion, while Snap23 knockdown in culture and in vivo mimicked the effects of ATF6 deletion on ANP secretion. These results define a new ATF6-ANP molecular signaling axis whereby ATF6 induces a non-canonical gene program required for regulated secretion to maintain cardiac myocyte proteostasis (Figure 2, Steps 11 and 12). Moreover, since Snap23 is required for regulated secretion of other neurotransmitters and peptides [124], it is likely that ATF6 serves a more widespread, required role in the regulated secretion of neurotransmitters and peptides. 

### 5.4. ATF6 Induces Stimulus-Specific Gene Programs

Over the course of studying ATF6 in various types of CVD, it was found that ATF6 be activated by diverse stimuli, not just misfolded proteins in the ER. These diverse ATF6 activators include oxidative stress and growth stimuli, each of which impact global proteome integrity, not just the ER proteome. Remarkably, during these various pathophysiological maneuvers, ATF6 activates unique gene programs, depending on the stimulus, and these programs serve stress-specific adaptive effects [50]. For example, oxidative stress results in ATF6-dependent induction of antioxidants, e.g., catalase, but growth regulators, e.g., Rheb. In contrast, growth stimuli activate growth regulators, but antioxidants. ATF6 gene deletion ablated the capacity for stimulus-specific induction of these genes and promoter analysis demonstrated that ATF6 bound to consensus ERSEs in a stimulus-specific manner [27,50]. A series of studies have been published highlighting a novel role for the secreted extracellular matrix protein, Thrombospondin 4, in serving as an escort protein for ATF6 activation by competing with GRP78 for binding to ATF6′s luminal domain and facilitating its the trafficking and subsequent processing. Overexpression of Thrombospondin 4 leads to ATF6 activation and confers protection in models of AMI and pressure-overload via the adaptive gene panel induced by ATF6 consisting of genes involved in membrane expansion allowing for enhancing protein and vesicular trafficking [125]. This finding is further supported by work in model cell lines that demonstrate that ATF6 can be activated by sphingolipids (e.g., dihydrosphingosine and dihydroceramide) without evidence for increased protein folding demand or protein misfolding and activate a unique gene profile allowing for homeostatic membrane expansion [126]. A further surprising finding of this study was that the activation mechanism in response to lipid accumulation was by virtue of a separate domain than that required for activation in response to the accumulation of misfolded proteins implicating, for the first time, specific domains of ATF6 required to activate downstream stimulus-specific gene programs. These data suggest that ATF6 is uniquely suited as a rapid sensor and responder to specific stress stimuli and capable of dictating genetic cellular reprogramming aimed at maintaining global proteostasis.

## 6. Small Molecule ATF6-Activators Confer Protection Against Cardiovascular Disease

**Figure 4:**
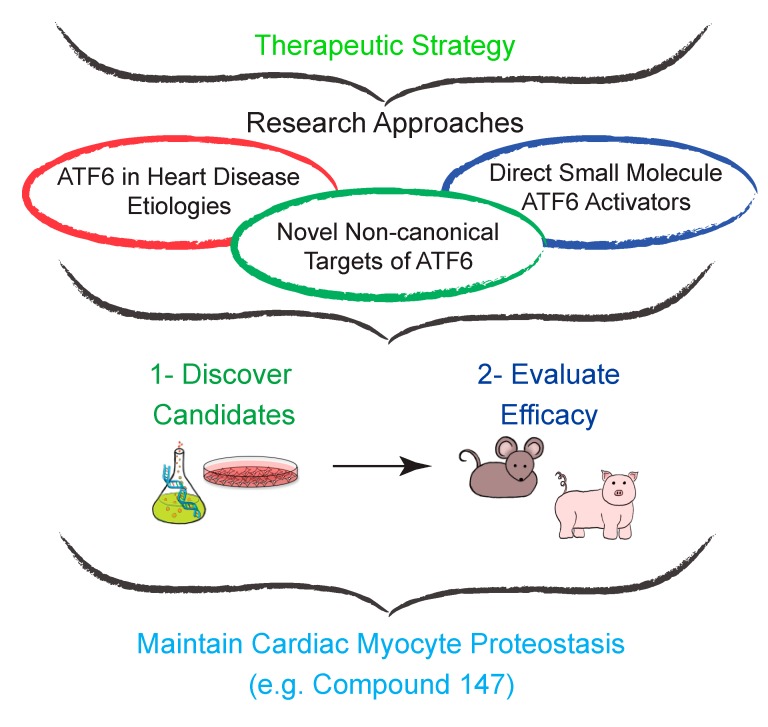
Approach to developing novel ATF6-based therapeutics. The therapeutic framework for developing ATF6-based therapeutics is conceptually simple in design. Three main research approaches are prioritized: (**1**) Expanding the scope of experimental animal models of various etiologies of heart disease in which ATF6 is studied. (**2**) Discovering other non-canonical targets of ATF6. (**3**) Using chemical biology to identify potent and specific small molecule activators of ATF6. Coordinately, these research approaches will converge into a streamlined experimental approach of (**1**) preliminary testing of lead small molecule activators in cell models of disease, in vitro, and (**2**) evaluating efficacy of these lead small molecule activators in small and large animal models, in vivo.

As described throughout this review, maintaining cardiac myocyte proteostasis is vital for cellular viability and function, and ATF6 has demonstrated efficacy as a therapeutic target for CVD and cardiac hypertrophy [27,50,116]. Thus, a conceptual framework with specific research approaches was designed in an attempt to identify key proteostasis regulatory pathways via discovering non-canonical gene targets of ATF6 using diverse animal models of various etiologies contributing to CVD. In coordination, lead candidate direct small molecule activators of ATF6 would be identified and validated for efficacy in small and large animal models of CVD and heart failure (Figure 4). However, small molecules, as regulators of transcription factors, have been an understudied topic of chemical biology, mainly due to fear of non-selectivity or lack of efficaciousness when interfering with transcriptional regulation [127]. Furthermore, targeting one of the three primary effectors of the ER UPR presents complications due to ambiguity concerning precise activation mechanisms and absence of known small molecule-binding sites [97]. However, recent studies identifying key methods of ATF6 activation have demonstrated the possibility of directing small molecule activators to these steps in the activation process (Figure 2, Steps 1 and 2).

Using a high throughput cell-based screen followed by medium-throughput transcriptional profiling and high-stringency filtering of a **644,971**-compound small molecule library, several non-toxic molecules that activate ATF6 were found, one of which is named compound **147** [128]. Compound **147** selectively activates ATF6, without effecting other UPR pathways, even in the absence of ER stress. An inhibitor of S1P, the Golgi protease that cleaves and activates ATF6, inhibited **147**-mediated ATF6 target gene induction. Using “click” chemistry and forms of **147** with chemical handles, the **147** interactome was defined in an attempt to demonstrate the mechanism by which it activates ATF6 [129]. It was discovered that **147** binds PDIs, which regulate disulfide bond formation in the ER. The mechanism of activation of ATF6 involves the dissociation of ATF6 oligomers in the ER to form ATF6 monomers that are able to relocate to the Golgi where S1P and S2P cleave ATF6, resulting in its activation. Compound **147** inhibits a unique group of PDIs that reside in a complex with ATF6 in the ER, where they maintain ATF6 in its inactive state. In this study, it was shown that **147** facilitates the movement of ATF6 out of the ER via PDI inhibition and subsequent dissociation from ATF6. 

Given the robust protection, ATF6 confers during AMI and post-AMI remodeling, the effects of pharmacological activation of ATF6 with **147** in a mouse model of reperfusion damage was chosen for initial efficacy testing [116]. Intravenous administration of **147** concurrently with reperfusion robustly and selectively activated ATF6 and downstream genes of the ATF6 gene program, protected the heart from AMI injury, preserved cardiac function, and decreased infarct size when assessed 24 h after drug administration/reperfusion. However, this protection was lost in ATF6 cKO mouse hearts. Thus, one dose of **147** concurrent with reperfusion was sufficient to induce the adaptive ATF6 gene program and provide protection from reperfusion damage during the first 24 h after AMI. Cardioprotection and ameliorated post-AMI cardiac hypertrophy was also observed in a similar experiment where heart damage and function were examined seven days after drug/reperfusion. Moreover, **147** had no deleterious effects in the absence of pathology, or in other tissues that were unaffected by reperfusion injury, an indicator of its safety. In fact, in the absence of AMI, **147** improved cardiac basal performance. This improvement was associated with the ability of just one administration of **147** to increase SERCA2a expression, resulting in improved Ca^2+^ uptake. Remarkably, by activating ATF6, **147** protected other tissues, including the brain, kidney, and liver, when they were subjected to maneuvers that induced reperfusion damage and impaired proteostasis. Moreover, administration of **147** every two to three days over a two-week timeframe had no untoward toxic effects in the heart, brain, liver, and pancreas. Thus, **147** selectively activates the ATF6 arm of the UPR in vivo, exhibiting significant potential as a therapeutic approach for treating AMI and reperfusion damage in a wide range of tissues [116].

## 7. Conclusions and Future Directions

Cardiac hypertrophy is an adaptive response to an increase in cardiac workload, either in a physiological or pathological manner. In order to maintain contractile function during growth, sarcomeric expansion in cardiac myocytes must be associated with proteostatic balance so as not to disrupt the integrity of the proteome with accumulation of toxic misfolded protein aggregates. ATF6 has been shown to be a primary adaptive sensor and responder to cardiac hypertrophy. In this setting, ATF6 induces both canonical and non-canonical gene panels associated with the balance of protein synthesis, folding, trafficking, and degradation. The recently demonstrated ability of ATF6 to be rapidly and transiently activated to induce adaptive genes specific to the stimulus or stressor has garnered great enthusiasm as a prime target for small molecule-based activators. Until recently, ATF6 has been a part of a class of proteins previously believed to be “undruggable”, but with research efforts detailing the mechanism of activation of ATF6 and stringent assays of small molecule library screening, the identification of ATF6-based therapeutics has taken great strides and shown promising efficacy in small animal models of CVD and other systemic proteostasis-based diseases (e.g., Compound **147**).

Still, ongoing research efforts must be aimed at understanding the mechanism of activation and action of ATF6 during various etiologies of CVD, so as to better design small molecules and better predict possible untoward effects of chronic ATF6 activation. One of the biggest questions remaining is the mechanism by which ATF6 chooses the gene program it influences during various pathologies. All non-canonical genes discovered to date that ATF6 induces in a stimulus-specific manner possess canonical ATF6-binding motifs in the proximal promoter region (ERSEs) [50]. ATF6 has been known to dimerize with other transcription factors as part of its transcriptional engagement including: SRF (serum response factor), Nrf1 (nuclear respiratory factor 1), PGC1α and β (peroxisome proliferator-activated receptor gamma coactivator 1-alpha and -beta), and ERRγ (estrogen-related receptor gamma) [104,110,130,131]. It would be enlightening to understand the dynamics of the nuclear ATF6-interactome in response to these various pathophysiological stimuli.

Furthermore, the finding that the expression levels of ATF6 and other essential components of the adaptive UPR decrease as a function of age, while the propensity for developing cardiac pathology increases as a function of age has highlighted a glaring need for continued studies of ATF6 function. The age-dependent decline in ATF6 has led to the exploration of therapeutic approaches aimed at enhancing ATF6 activity in the aged, pathologic heart in hopes of improving proteostasis thereby enhancing cardiac myocyte contractility, and reducing the progression to heart failure characterized by the accumulation proteotoxic aggregates, fibrosis, and decreased cardiac compliance. 

The development of proteostasis- and ATF6-based therapeutics is still in its infancy and reflecting on the incredible advancement in developing small molecule activators in a relatively short period of time provides a great deal of optimism as the field of proteostasis continues to develop. Hopefully, compounds like **147** will act as catalysts for the design of future studies aimed at targeting the UPR for treating CVD and other systemic diseases.
